# Functional bionanomaterials for cell surface engineering in cancer immunotherapy

**DOI:** 10.1063/5.0045945

**Published:** 2021-05-03

**Authors:** Sheng Ma, Yudi Xu, Wantong Song

**Affiliations:** 1 Key Laboratory of Polymer Ecomaterials, Changchun Institute of Applied Chemistry, Chinese Academy of Sciences, Changchun 130022, China; 2 Jilin Biomedical Polymers Engineering Laboratory, Changchun 130022, China; 3 University of Chinese Academy of Sciences, Beijing 100039, China

## Abstract

The cell surface is the forward position in cancer immunotherapy, with surface ligand and receptor interactions between various cells for determining immune privilege or recognition. Therefore, cell surface engineering (CSE) that manipulates the surface interactions between the immune effector cells (IECs) and tumor cells represents a promising means for eliciting effective anticancer immunity. Specifically, taking advantage of the development in biomaterials and nanotechnology, the use of functional bionanomaterials for CSE is attracting more and more attention in recent years. Rationally designed functional biomaterials have been applied to construct artificial functional modules on the surface of cells through genetic engineering, metabolic labeling, chemical conjugation, hydrophobic insertion, and many other means, and the CSE process can be performed both *ex vivo* and *in vivo*, on either IECs or tumor cells, and results in enhanced anticancer immunity and various new cancer immunity paradigms. In this review, we will summarize the recent exciting progresses made in the application of functional bionanomaterials for CSE especially in establishing effective recognition and interaction between IECs and tumor cells.

## I. CELL SURFACE ENGINEEING IN CANCER IMMUNOTHERAPY

The cell membrane functions as more than just mechanical support and protection for cells.^1^ The cell membrane is also involved in the communications between different cells as well as the communications between cells and the extracellular environment.^2^ Such communications mainly rely on interactions between receptors and ligands expressed on the cell surface. Thousands of biomolecules, mainly proteins and glycans, are expressed on the cell membrane, which functions for recognizing by other cells as well as capturing and sensing biochemical molecules or signals from the surroundings.^3^ It has been widely appreciated that cell-to-cell interactions through direct cell membrane contact is associated with various physiological processes such as immune recognition and immune elimination.^4^ Manipulating cell surface properties by regulating functional biomolecules expressed on the cell surface can change the fate of cells and regulate cells involved in physiological processes.^5–11^ *This is in particular the case in cancer immunotherapy, since the surface recognitions and interactions between immune effector cells* (*IECs) and tumor cells are the central scenario of immunotherapy.*^12–14^ The therapeutic efficacy of IECs including T cells, natural killer (NK) cells, and macrophages depends on the strength and specificity of the interactions between the receptors and ligands on these IECs and the targeted tumor cells.^15,16^ As a result, various strategies have been invented for modulating the surface interactions to strengthen or weaken the recognitions and proved to be meaningful in cancer immunotherapy. For example, monoclonal antibodies against programmed cell death protein 1 (PD-1)/programmed death-ligand 1 (PD-L1) have been applied to regulate the function of immune checkpoint proteins expressed on T cell and tumor cell membranes for relieving the negative immune regulation and recovering the activity of T cells to tumor cells.^17,18^ Antibodies against other cell surface proteins such as CD47 and sialic acid binding immunoglobulin lectins (Siglecs) have also been developed for priming an effective antitumor immune responses.^19–23^

From another aspect, directly manipulating the IEC or tumor cell surfaces with various cell surface engineering (CSE) approaches for constructing artificial receptors or ligands on the surface represents an alternative promising strategy for adjusting the immune recognition process in antitumor immunity. These approaches have shown promising aspect in developing new therapeutic strategies for cancer immunotherapy. Many exiting progresses have come out from this aspect recently, and some of them have been proved to be quite successful in clinical studies.^7^ For example, the chimeric antigen receptor (CAR)-T cells are fabricated through genetic engineering of isolated autologous T cells *ex vivo* to express CARs on the cell surface and re-infused back to patients similar to a blood transfusion for tumor cell-specific recognition.^24^ Up to now, three kinds of CAR-T cell products have entered the market and more are in the clinical trials.^25–29^ Besides genetic engineering, CSE could also be performed with methods including metabolic labeling,^30–32^ chemical conjugation,^33^ hydrophobic insertion,^34^ and many others.^35^ *Importantly, to construct recognition molecules more precisely and intelligently, many functional biomaterials, including synthetic polymers, proteins, nucleic acids and inorganic materials, have been utilized for CSE.*^36–39^ Functional biomaterials have demonstrated great potential and excellent application scalability for CSE as they can adjust the compositions and functions of materials according to the requirements. Compared with genetic engineering approach, re-engineering cellular interfaces with natural or synthetic functional biomaterials will enable intelligent design with stimuli-responsive properties or many other non-natural functions. These properties provide a great empowerment in cancer immunotherapy and give birth to many new cancer immunotherapeutic paradigms. In this review, we will give a short summary on the recent progresses made in designing functional bionanomaterials for CSE in cancer immunotherapy. Specifically, this review will focus on using bionanomaterials for reestablishment of the specific recognition and interaction between IECs and tumor cells, and enhancing the tumor killing capability of IECs. There are many other reviews on the broad topic of CSE, including cell membrane bioconjugation, non-genetic engineering of cells, engineering cell membranes for inflammation, cell membrane-derived nanomaterials, and so on. We refer the interested readers to the other excellent recent reviews.^40–45^

## II. FUNCTIONAL BIOMATERIALS

Functional biomaterials are materials designed with intelligent properties which could respond to the biological environment or provide specific bioactive signals during the practical application.^46–48^ Functional biomaterials have been widely used for disease treatment, diagnosis, cell culture, and tissue repairment because of their intelligence and versatility.^49,50^ According to the composition of the materials, functional biomaterials can be divided into organic/polymeric materials, inorganic materials, as well as organic–inorganic hybrid materials [e.g., metal-organic framework (MOF)]. The most potent advantage of functional biomaterials in antitumor therapy lies in that under specific *in vivo* stimulations, their physical or chemical properties can switch from one state to another, and drugs or other therapeutic agents could be released from these formulations in a timely or spatial controllable manner.^51,52^ These properties enabled a wide application of functional biomaterials for designing intelligent nanomedicines for cancer management.^53–57^

### A. Organic/polymeric functional biomaterials for cancer management

**FIG. 1.**
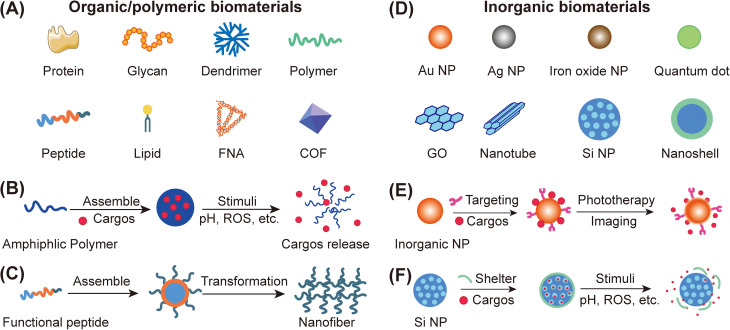
Classification of biomaterials and schematic depicting intelligent design for drug delivery. (a) Main types of organic/polymeric biomaterials. (b) Schematic of amphiphilic polymeric biomaterials loading cargos and realizing cargos release under specific stimuli. (c) Schematic of functional peptide assembling in to nanoparticles (NPs) *in vitro* and transforming into nanofiber in tumor tissues. (d) Main types of inorganic biomaterials. (e) Schematic of inorganic nanoparticles modified with target molecules and loading cargos for phototherapy or imaging. (f) Schematic of the preparation of mesoporous silica nanoparticles with surface shielding and loading cargos, and realizing cargos release under specific stimuli. FNA: framework nucleic acid; COF: covalent organic framework; ROS: reactive oxygen species; GO: graphene oxide; NP: nanoparticle.

Organic/polymeric functional biomaterials include protein and glycan, synthetic dendrimer, polymers, peptides, lipids, framework nucleic acids (FNAs), covalent organic frameworks (COFs), etc. [Fig. 1(a)].^58–66^ These organic biomaterials exhibit excellent biocompatibility and most of them can be degraded *in vivo* or eliminated from the body, which lends them significant potential for clinical translation.^67^ For example, natural albumin, the most important protein in plasma, has been used as a delivery carrier to address insolubility and dosage limitations encountered with paclitaxel.^68^ Compared with natural organic biomaterials, the structures and properties of synthetic organic biomaterials can be more precisely designed.^69,70^ Amphiphilic or completely hydrophilic synthetic polymers are ideal materials for the delivery of drugs, genes, proteins, and nucleic acids by physical entrapment or chemical conjugation. The assembled nanostructures could enhance *in vivo* stability, prolong blood circulation time, and improve tumor accumulation by passive or active targeting strategies.^71^ In addition, stimuli-responsive linkers could be introduced into organic biomaterials to control the release behavior of the loaded cargos triggered by specific stimuli, such as low pH, high reactive oxygen species (ROS), and exogenous stimulation including irradiation, ultrasound, and light [Fig. 1(b)].^72–74^ Some organic biomaterials can be designed to reverse the surface charge or assembly sizes in response to specific stimuli in the tumor tissue, thus resulted in enhanced penetration and uptake by tumor cells;^75–77^ others can be designed as programmable entities for automatic transformation *in vivo* to realize pre-designed assembly/disassembly for intelligent drug delivery [Fig. 1(c)].^53,78–81^

### B. Inorganic functional biomaterials for cancer management

Inorganic biomaterials, including metal-based biomaterials, silicon-based biomaterials, and carbon biomaterials, have been extensively studied for radiotherapy, phototherapy, magnetic resonance imaging, and drug delivery in cancer management due to their inherent superior physicochemical properties (including optical, thermal, catalytic, and magnetic properties) [Fig. 1(d)].^82^ Metal-based biomaterials include gold nanoparticles, silver nanoparticles, metal oxide nanoparticles, and metal hybrid nanoparticles, which can be functionalized by introducing shelter, targeting molecules, and loading cargos like nucleic acid, fluorescent molecules, and drugs.^83–86^ They have been applied in cancer diagnosis and phototherapy, especially near-infrared region (NIR) phototherapy and radiotherapy due to adjustable magnetic and optical resonance properties [Fig. 1(e)].^87,88^ Carbon nanomaterials, including nanographene sheets and carbon nanotubes, have been widely applied in biomedical application including cancer treatment.^89,90^ Nanographene sheets are composed mainly of graphene and its derivatives such as graphene oxide (GO), reduced graphene oxide (rGO), and GO-nanocomposites, which all exhibit excellent NIR photothermal conversion efficiency.^91^ Mesoporous silica biomaterials are another kind of important inorganic biomaterials, which can be used for drug, protein, and photosensitizer delivery due to their adjustable mesoporous size.^92^ With abundant available reactive groups on the surface of mesoporous silica biomaterials, these mesoporous silica biomaterials can integrate optical, magnetic, and electronic properties for cancer diagnosis and treatment.^93^ Hydrophilic materials can also be introduced into the assembly structure of these mesoporous silica biomaterials as shelter to prolong *in vivo* circulation. Moreover, various stimuli-sensitive pore blockers, such as metal nanoparticles and organic molecules, have been decorated on the surfaces of mesoporous silica nanoparticles to control the release of loaded cargos in response to external stimuli [Fig. 1(f)].^94,95^

## III. REPRESENTATIVE EXAMPLES OF USING FUNCTIONAL BIOMATERIALS FOR CSE IN CANCER IMMUNOTHERAPY

The application of functional biomaterials for CSE in immunotherapy can be performed from two aspects: *ex vivo* and *in vivo*. For *ex vivo* CSE, functional biomaterials are mainly used for gene-editing or directly engineering of the isolated IECs for improving the recognition ability and therapeutic effects after adoptively transferred back to the patients. For *in vivo* CSE, functional biomaterials can be applied to directly manipulating the IECs with empowered ability, or engineering the tumor cells for easy recognition by the IECs.

### A. Functional biomaterials for ex vivo CSE

**FIG. 2.**
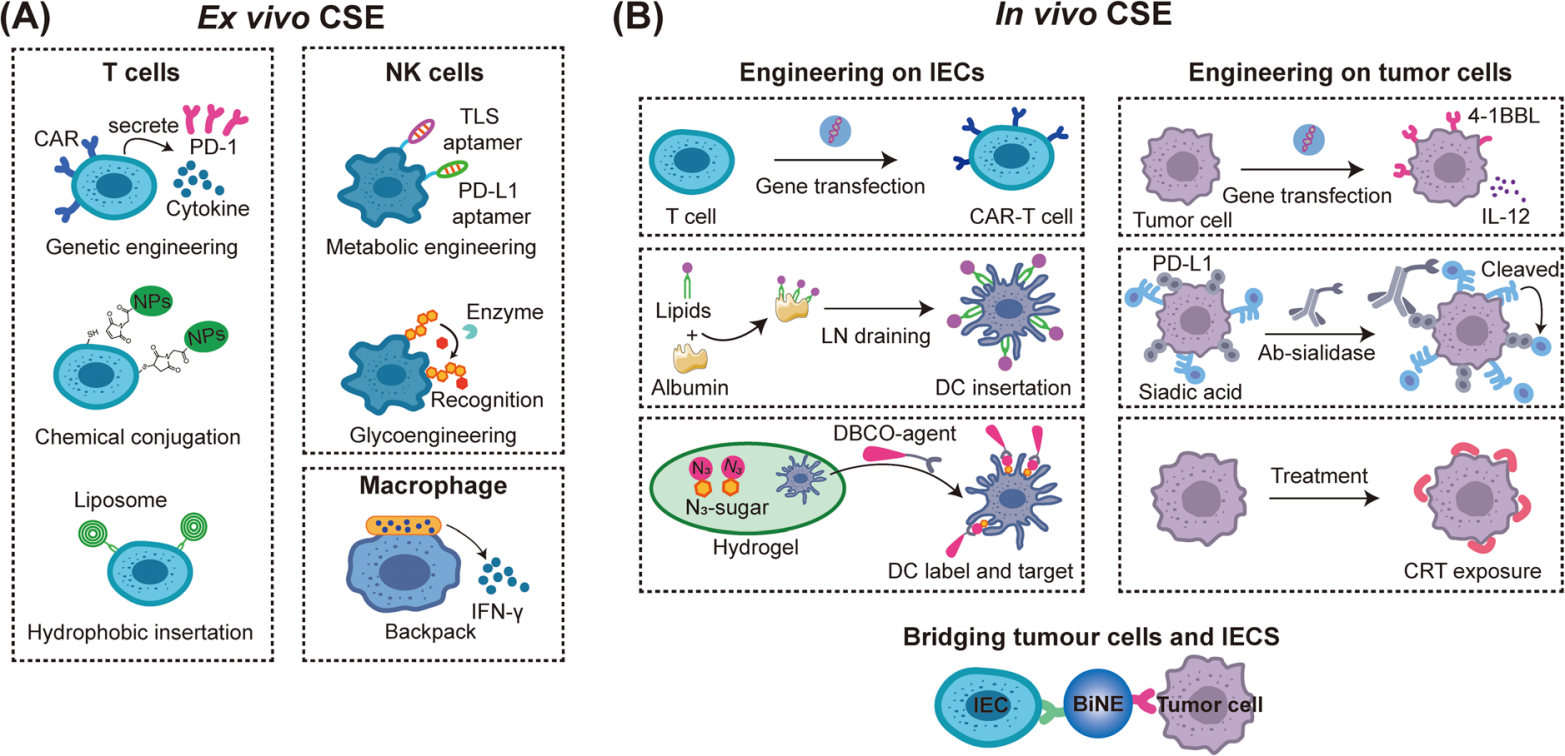
Overview of approaches currently used in *ex vivo* and *in vivo* CSE with functional biomaterials for cancer immunotherapy. (a) Main types of *ex vivo* immune cell surface engineering with functional biomaterials. The CSE approaches of T cells can be divided into three kinds: (1) genetic engineering T cells; (2) covalently conjugating CAR-T cell surface thiols with maleimide containing nanoparticles; and (3) hydrophobic inserting functional liposome into T cells surface. The CSE approaches of NK cells can be divided into two kinds: (1) aptamer equipping NK cells through metabolic engineering and (2) glycoengineering NK cell membrane with glycan ligands under the catalysis of enzyme. As for macrophages, shape-anisotropic particles have been used to backpack macrophages for CSE. (b) Main types of *in vivo* immune cell and tumor cell surface engineering with functional biomaterials. *In vivo* immune cell surface engineering can be achieved by constructing CAR-T cells *in vivo* with gene carriers to transfect circulating T cells or with amphiphile CAR-T ligands inserting dendritic cells (DCs) as *in situ* CAR-T vaccine for CAR-T cells boosting. *In situ* metabolic labeling of DCs and subsequent targeting delivery of agents via biorthogonal reaction represents another successful example. As for *in vivo* tumor cell surface engineering, *in situ* genetic engineering tumor cells to express co-stimulatory molecules (4-1BBL) and secrete immunostimulatory cytokines (IL-12), targeted desialylation with antibody–sialidase conjugates and changing the protein presented on tumor cell surface through biomaterial mediated treatments have achieved great progress in recognition by IECs. Besides, bispecific nano-bioconjugate engager (BiNE) has also been used for bridging IEC and tumor cell *in vivo*. LN: lymph nodes; Ab: antibody; BiNE: bispecific nano-bioconjugate engager.

*Ex vivo* CSE for immunotherapy mainly works on improving the ability of IECs to recognize cancer cells or overcome obstacles that IECs face in the tumor microenvironment which has been used for T cells, NK cells, and macrophages [Fig. 2(a)].^96,97^ For example, genetic engineering of immune cells has been widely applied in adoptive cell therapy (ACT) including CAR-T cells, CAR-NK cells, and CAR-macrophages.^98–100^ Immune cells are genetically engineered to express tumor antigen receptors on the cell surface and activated *in vitro* for adoptive transfer and tumor target therapy.^101^ Among all, CAR-T cell therapy has achieved great success in clinic especially in B cell lymphoma.^102,103^ However, current CAR-T cell therapy shows limited efficacy against solid tumors, partially because of the immuno-suppressive microenvironment in the tumor tissues.^104^ Aiming at this problem, several strategies such as “armoured” CAR-T cells by engineering T cells simultaneously expressing CAR and other immune checkpoint blockade fragments or secreting cytokines have been proposed.^105–107^ The progress in these genetic engineering techniques has brought abundant development to the ACT therapy.

Using functional biomaterials or assemblies for direct cell surface decoration of the IECs represents another direction for empowering the ACT therapy.^108^ Compared to the gene-editing method, use of biomaterials for non-genetic decoration is much safer and easier, thus has attracted much research interest in recent years. For example, Irvine's group first proposed the strategy of utilization of the thiol groups on the T cell surface for surface chemical conjugation of synthetic nanoparticles containing IL-15Sa and IL-21 to cooperatively promote T cell function *in vivo*. In a metastatic B16F10 melanoma model, such kind of cytokine containing nanoparticles back-packed T cells resulted in significant antitumor efficacy.^33^ Similar strategies have also been applied in T cells for carrying many other therapeutic cargos.^35,109–111^ Hydrophobic insertion of cell membrane represents another strategy for CSE of T cells with nanoparticles by inserting lipid tails into cell membrane. This strategy is relatively easy without affecting the function of the modified cells. For example, Hao *et al.* used two-tailed lipids to anchor a liposome with avasimibe on the T cell membrane through hydrophobic insertion and a biorthogonal reaction. The loaded avasimibe could be retained on the T cell surface during circulation while locally released in the tumor tissue to induce rapid T cell receptor clustering and sustained T cell activation, so as to improve the therapeutic effect of adoptive T cells to the solid tumor.^34^ Besides T cells, NK cells and macrophages have also been functionalized with biomaterials for expanding their *in vivo* performance after adoptive transfer. For example, Zhang *et al.* proposed an aptamer-equipping strategy to generate specific, universal and permeable (SUPER) NK cells through metabolic glycan biosynthesis and biorthogonal click chemistry for enhancing NK cell therapy in solid tumors.^112^ NK cells can be potentially developed as off-the-shelf adoptive cellular therapy because of lacking the expression of major histocompatibility complex (MHC) class I molecules on the cell surface. However, NK cells generally lack inherent selectivity toward cancer cells and are known to be notoriously adverse to gene transfection. Therefore, using glycoengineering for equipment of NK cells with tumor targeting ligands represent an exciting new approach for cancer treatment.^113,114^ As for macrophages, Shields *et al.* utilized interferon-γ (IFN-γ) containing shape-anisotropic particles to back-pack macrophages. These backpacked macrophages expressed M1 marker at least within 48 h after systemic injection, and induced a shift in the polarization of tumor associated macrophages under the continuous stimulation of IFN-γ, resulting in a potentiated antitumor responses against 4T1 triple negative breast tumors.^115^ Although the starting point of these CSE technologies is to enhance the combination of cell surface with these exogenous biomaterials, the researchers have carefully adjusted the scheme in the research process to avoid affecting the viability of engineered cells.^33^

### B. Functional biomaterials for *in vivo* CSE

#### 1. Engineering on IECs

Compared with the complicated and high-cost *in vitro* CAR-T cell manufacturing procedures, *in vivo* CAR-T cells fabrication might be much easier and cheaper. Engineering circulating T cell surface with functional biomaterials is a promising alternative strategy, which can easily and quickly generate tumor specific T cells [Fig. 2(b)]. Utilizing functional biomaterials as gene transfection carriers to directly transfect circulating T cells for CAR expression represents a straightforward method. For example, Smith *et al.* realized *in vivo* leukemia-specific CAR-T cell generation with a simple nanostructure fabricated by cationic poly(β-amino ester) (PBAE), plasmid DNA encoding the leukemia-specific CAR, and polyglutamic acid conjugated with anti-CD3ε f(ab)_2_ fragments.^116^ These well-designed polymeric gene carriers could quickly recognize circulating T cells and efficiently introduce leukemia-targeting CARs on the T cell surface. Since polymer nanoparticles can be easily manufactured and stored, this method provides a practical “on-demand” setting for generating antitumor immunity. Different from the above method, Ma *et al.* proposed an *in situ* CAR-T vaccine boosting strategy by constructing amphiphile CAR-T ligands which chaperone with albumin after injection, trafficking the antigens to lymph nodes (LN) and anchoring the antigens to the antigen-presenting cell surface.^117^ Such amph-ligands, combined with CAR-T cell transfer, yielded CAR-T populations nearly 200-times-greater compared with CAR-T cell transfer alone. These persisting CAR-T cells are “younger” and more energetic, and thus animals receiving CAR-T combined with repeated amph-vaccine boosting significantly delayed tumor growth and prolonged the mice survival time. In another study, Wang *et al.* used an azido-sugars containing hydrogel to metabolically label dendritic cells (DCs) with azido groups *in situ*. The azido-labeled DCs could persist for weeks and further capture dibenzocyclooctyne (DBCO)-modified antigens or cytokines, thus improved the priming of antigen-specific CD8^+^ T cells.^118^

#### 2. Engineering on tumor cells

*In situ* engineering of tumor cell surface represents another direction for CSE in cancer immunotherapy. The aim of *in vivo* tumor CSE is to enhance the interactions between tumor cells and the IECs [Fig. 2(b)]. Nanoparticles with a size range between 20 and 200 nm tend to accumulate in tumor after injection due to the enhanced permeability and retention (EPR) effects,^119^ which provides an opportunity for targeted tumor cell surface modification *in vivo*. For example, Tzeng *et al.* used biodegradable gene-delivery nanoparticles for *in situ* genetic engineering of tumor cells to express co-stimulatory molecules (4–1BBL) on the cell surface.^120^ The tumor cells were simultaneously engineered to secrete immunostimulatory cytokines (IL-12), which along with 4–1BBL induced significant T cell-mediated cytotoxic immune responses in B16F10 and MC38 tumor models. Altered glycosylation has been regarded as a hallmark of malignancy and usually induces an immunosuppressive effect to IECs. Among them, the interaction between sialic acids and siglecs could serve as a glycol-immune checkpoint modulating the immune recognition between IECs and the tumor cells.^21,23^ Therefore, modulation of tumor cell surface glycans represents another promising direction in cancer immunotherapy. Bertozzi's group proposed to utilize trastuzumab–sialidase conjugates for selective degradation of sialylated glycans from HER2-positive breast cancer cells, which enhanced tumor cell susceptibility to antibody-dependent cell-mediated cytotoxicity (ADCC) and enhanced NK cell activity.^121^ The degradation of sialoglycans with antibody–sialidase conjugates represents a promising modality for glycol-immune checkpoint therapy.^122^

In addition to the above strategies, endogenous reprogramming the expression of tumor cell surface proteins by drug treatment is another method for enhancing the recognition of tumor cells by the IECs. For example, some treatments could induce immunogenic cell death (ICD) of cancer cells and increase the calreticulin exposure on the cell surface, which serves as a engulfment signal to promote antigen-presentation and recognition by the immune cell.^123,124^ We recently reported a tumor-specific enhanced oxidative stress polymer conjugate (TSEOP) for boosting oxidative stress and inducing ICD in tumor cells. Significant activation of the immune responses was observed and single usage of this polymer conjugate resulted in complete tumor eradication in two murine tumor models.^125^ Deng *et al.* utilized redox sensitive nanoparticles to transport endoplasmic reticulum targeting photosensitizer, which provoked an antitumor immune response by inducing the exposure of calreticulin to tumor cell surfaces serving as an “eat me” signal after irradiation.^126^

#### 3. Engaging of IECs and tumor cells

In addition to direct engineering of the IEC or tumor cell surfaces, bispecific nano-bioconjugate engager (BiNE) with the ability of bridging immune cells and tumor cells together represents another promising strategy to improve the recognition of immune cells to tumor cells *in situ* [Fig. 2(b)].^127^ The injected nano-engagers may first bind on the surface of either IECs or tumor cells based on the affinity of targeting moieties to these cells. Compared to the bispecific T cell engagers (BiTEs) or bispecific antibodies, the BiNEs could enable multivalent interactions and cargo loading for enhancing the functions of the immune cells.^128^ For example, Cheng *et al.* reported a type of synthetic multivalent antibodies retargeted exosomes (SMART-Exos) through genetically displaying CD3 and epidermal growth factor receptor (EGFR) antibodies on the exosome surface. The SMART-Exos could mediate the conjugation of T cells with tumor cells highly expressed with EGFR and enhance the killing effect of T cells on tumor cells.^129^ Yuan *et al.* prepared a multivalent bispecific nano-bioconjugate engager (mBiNE) by chemically conjugation of anti-human epidermal growth factor receptor 2 (HER2) antibody and calreticulin onto carboxylated polystyrene nanoparticles. The mBiNE stimulated HER2 targeted phagocytosis both *in vitro* and *in vivo* and produced durable antitumor immune responses against HER2-expressing tumors after injection.^130^ Similarly, Zhang *et al.* prepared a bispecific nanoparticle SNPA_CALR&aCD47_ by chemically conjugating anti-phagocytic signals CD47 antibody (aCD47) and pro-phagocytic molecule calreticulin on modified silica nanoparticles, and realized significantly promoted phagocytosis of macrophages on tumor cells *in vivo*.^131^ Similar to BiNE, tri-specific nano-engager has been developed by adding an antibody that can activate IECs. For example, Au *et al.* constructed a type of tri-specific nano-engager (α-EGFR/α-CD16/α-4–1BB nanoparticles) with EGFR antibodies for tumor cell targeting and CD16 and 4–1BB antibodies for NK cell recruitment and activation. This trispecific NK cell nano-engager can further load with chemo agents for inducing robust chemoimmunotherapy *in vivo*.^132^

## IV. CONCLUSION AND FUTURE PERSPECTIVES

The cell is the basic unit of an organism, and cell–cell interactions through surface molecules is the basic phenomenon in an organism. As a result, redirecting the IECs to recognize and act on the tumor cells constitutes the core event in cancer immunotherapy. This redirecting process can be accomplished by injecting antibodies to block some negative regulations (like anti-PD-1/PD-L1) or by directly engineering on the surface of IECs or tumor cells. Specifically, CSE to artificially improve the ability of cell recognition as well as the intensity and frequency of cell–cell interaction represents a promising new direction for cancer immunotherapy. Currently, the major method for CSE in cancer immunotherapy is viral vector-based gene engineering on the isolated autogenous T cells. Although the representative products like CAR-T cells have entered the market, viral vector-based transfection still faces risks for integration into the host's genome, and the transfection efficiency is quite low to other immune cells like NK cells or macrophages.

**FIG. 3.**
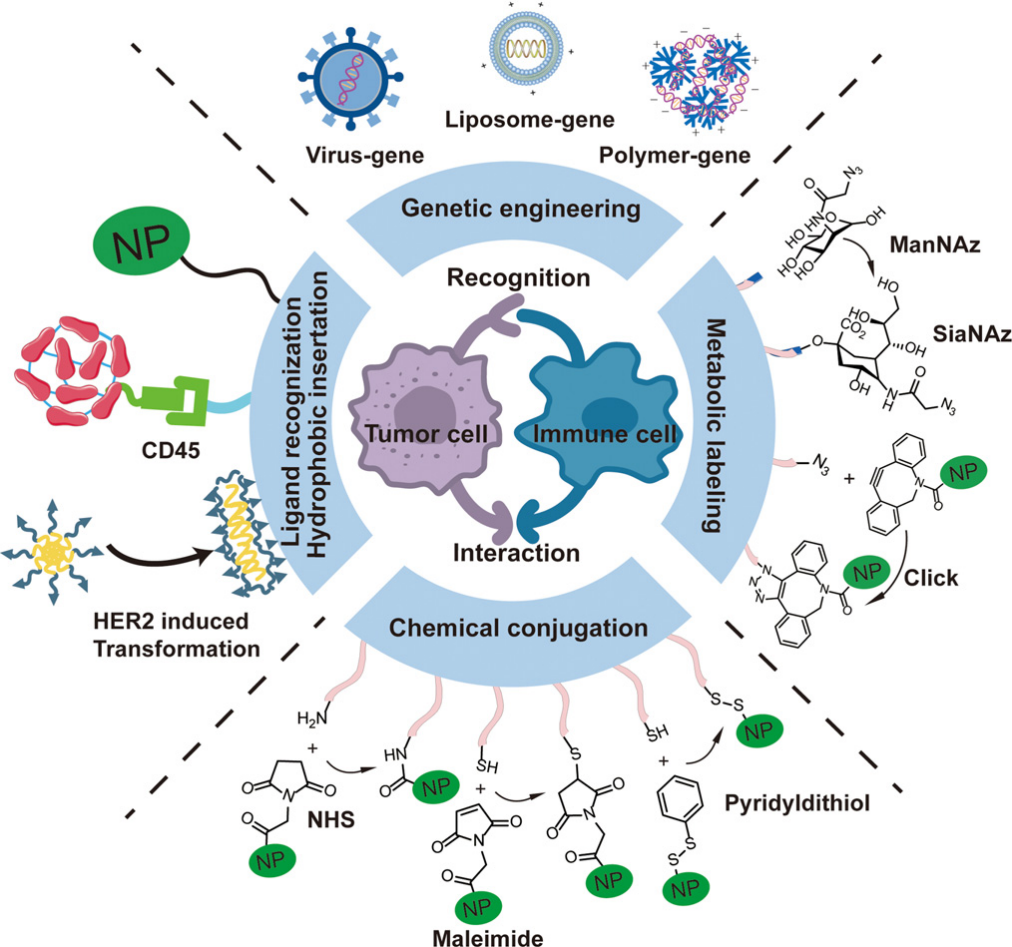
Cell surface engineering techniques for modulating recognition or interaction between tumor cells and immune cells in cancer immunotherapy. ManNAz: *N*-α-azidoacetyl mannosamine; SiaNAz: *N*-α-azidoacetyl sialic acid; NHS: *N*-hydroxyl-succinimidyl ester; NP: nanoparticle; HER2: human epidermal growth factor receptor 2.

The integration of functional bionanomaterials and CSE opened up a broad new research field for bioengineering due to the versatility, intelligence, and diversity in designing bionanomaterials. In this review, we summarized the recent progress in using functional bionanomaterials for CSE in cancer immunotherapy, including both *in vitro* and *in vivo* means. Generally, the current CSE methods could be classified into the following four aspects and we believe there is still much room for improvement (Fig. 3). (1) Genetic engineering: Genetic engineering as a powerful technique to regulate cell surface proteins can produce long-term cell surface modification. However, the genetic engineering method is not available to all types of cells due to the difficulties in transfection, and permanent genetic modification may possess long-term side effects. Although viral vector is widely used in current gene transfection, potential problems such as uncontrollable gene expression and immune risk related to the virus vector still exist in CAR-T producing procedures.^133^ More than that, viral vectors have a limited DNA cargo size (typically < 10 kb), which limits inclusion of advanced engineering designs to improve CAR-T cell targeting, function, trafficking, and persistence.^43^ Non-viral gene carriers based on functional biomaterials, including lipid, cationic polymers, aptamers, and inorganic carriers, have been explored given their high gene-loading capacity, ease of preparation, and specific cell gene engineering.^134,135^ These biomaterials are usually positively charged and co-assembled with DNA or RNA by electrostatic interactions. However, reports thereof remain sparse in terms of the utilization of biomaterials for CAR-T cell gene engineering, which might be due to the low transfection efficiency of functional biomaterials on T cells. More efforts should be devoted to developing functional biomaterials that are more suitable for T cell and other immune cells transfection. (2) Metabolic labeling: Metabolic labeling can easily introduce chemical reactive groups to cell surface glycoproteins at the required density through natural carbohydrate biosynthetic pathways. Pioneering work by Bertozzi provides a versatile method for CSE with various of functional groups by metabolic oligosaccharide engineering (MOE).^32^ Unnatural azido-*N*-acetylmannosamine (ManNAz) or azido-*N*-acetylneuraminic acid (NeuNAz) could be decorated on the cell surface through the sialic acid biosynthesis pathway and provides many reactive azido groups on the cell surface. This enabled post-modification with various molecules or nanoparticles through click reactions with dibenzocyclooctyne (DBCO).^136^ Other reactions could also be applied in this process and the CSE could be performed *in vivo* via tumor cell selective biorthogonal reactions.^137^ (3) Chemical conjugation: Because of the existence of amine or thiol groups on the surface of many IECs, chemical conjugation of biomolecules or nanoparticles onto the cell surface with reactions between *N*-hydroxysuccinimide or maleimide and these groups is a straightforward method for CSE on IECs. One factor that may influence the efficiency of this method is the surface amine or thiol densities may change with the status of the cells. For example, activated T cells show enhanced thiol groups on the surface. Therefore, much effort still needs to be made to investigate the surface properties of cells to ensure standardization of this method. (4) Hydrophobic insertion, ligand recognition, or surface assembly: Based on the natural components of the cell membranes, using lipids or cholesterol to anchor biomolecules or nanoparticles directly on the cell surface is simpler and more convenient to use compared with other CSE technologies. However, it needs to mention that hydrophobic insertion is not quite stable and the loaded cargos may be lost during circulation. Ligand recognition or surface assembly may be alternative methods for enhancing the stability of the cargos on the cell surface. For example, Zhang *et al.* designed an intelligent supramolecular peptide, BP-FFVLK-YCDGFYACYMDV (TMP1), in which the BP and FFVLK act as the hydrophobic core and YCDGFYACYMDV binds to HER2 expressed on the tumor cell surface.^138^ This supramolecular peptide could self-assemble into nanoparticles in an aqueous environment and maintain its nanostructure in blood circulation, while once bonded with HER2, it automatically transformed into a nanofibrous structure on the cell surface. This interesting design could be used for *in situ* CSE with functional groups for the immune recognition. Overall, we believe this research direction is still in its infancy and large opportunity exists in this inter-disciplinary field.

## Data Availability

Data sharing is not applicable to this article as no new data were created or analyzed in this study.
